# Positive Instruction in Music Studios: Introducing a New Model for Teaching Studio Music in Schools Based upon Positive Psychology

**DOI:** 10.1186/s13612-015-0036-9

**Published:** 2015-10-26

**Authors:** Tim Patston, Lea Waters

**Affiliations:** Graduate School of Education, University of Melbourne, Melbourne, Australia; Geelong Grammar School, Victoria, Australia; Centre for Positive Psychology, Graduate School of Education, University of Melbourne, Melbourne, Australia

**Keywords:** Positive psychology, Studio music instruction, Process praise, Character strengths, Music pedagogy

## Abstract

This practice paper explores the intersection of school studio-music pedagogy and positive psychology in order to enhance students’ learning and engagement. The paper has a practitioner focus and puts forward a new model of studio teaching, the *Positive Instruction in Music Studios* (PIMS) model that guides teachers through four key positive psychology processes that can be used in a music lesson: positive priming, strengths spotting, positive pause, and process praise. The model provides a new, positively oriented approach to studio-music pedagogy that can be integrated into specific methods-based programs to enhance student learning and engagement.

## Background

Positive psychology (PP) is a relatively new field (Duckworth et al. 2005; Linley et al. 2006; Seligman and Csikszentmihalyi 2000) that scientifically studies the flourishing and optimal functioning of individuals, groups, and institutions (Gable and Haidt 2005; Linley et al. 2006). It focuses on the strengths, virtues, beneficial conditions, and processes that contribute to well-being and positive functioning. Positive psychology aims to expand the focus of psychology from a preoccupation with repairing the negatives in life to also extending the positives (Seligman and Csikszentmihalyi 2000). Since its inception, the field of PP has grown rapidly and has expanded beyond the field of psychology into disciplines such as education (Rusk and Waters 2013).

The application of PP to education has been termed “positive education” by Seligman et al. (2009), who define positive education as an approach to education that fosters traditional academic skills and skills for happiness/well-being. Researchers and practitioners in the positive education movement argue that academic achievement and well-being do not need to be treated as mutually exclusive agendas within schools. Schools can “teach both the skills of well-being and the skills of achievement” (Seligman et al. 2009, p. 294) without compromising either. Positive education seeks to ensure that a student’s academic abilities are developed in unison with his/her character development and resilience (Bereznicki et al. 2008; Berkowitz 2005; Vaughan 2000). Waters (2011), White and Waters (2014), and Pawelski and Moore (2012) contended that students can be exposed to PP through the inclusion of positive models and teaching practices in the curriculum of traditional academic disciplines. In line with this claim, we suggest that music studio teaching could be a pedagogy through which PP is infused.

As such, the aim of this practice paper is to critically analyse the current research literature, translate science to practice, and outline a new framework of music studio teaching, the *Positive Instruction in Music Studios* (PIMS) model. This model is based upon the new science of PP and seeks to support the teacher in using differentiated teaching and learning approaches, making the learning visible, forming positive teacher–student relationships, and promoting student well-being.

## The Capacity for Music Education to Boost Student Well-being

According to Hallam (2001), music education has the capacity to enhance a student’s intellectual, social, and emotional development by building engagement, self-confidence, concentration, and emotional sensitivity. Custodero (2003) argued that music is a way to encourage intrinsic motivation and engagement in students. In this regard, music may be a discipline area that aligns well with the intentions of PP and positive education. Rich (2012) contended that PP practices can play a role in the development of students’ talent by fostering flow, creativity, and resilience. More specifically, he argued that “a strengths-based approach that reflects an understanding of the conditions of flow may lead to… resilience and joy for the musician” (p. 1164). Rich also maintained that PP can facilitate the evolution of more positive models of talent development in music.

However, the study of PP in music education is sparse, and research shows that music students often suffer high levels of stress and anxiety (Fehm and Schmidt 2006; Maroon 2002; Osborne and Kenny 2005; Patston 2014). As such, interventions in music education have typically focused on how to reduce these negative emotional states using techniques that involve stress reduction and cognitive behavioural therapy (Craske and Craig 1984; Patston 2014; Roland 1993) rather than on systematically promoting positive states in music students.

This notwithstanding, the modest amount of research into PP in music education completed so far has been promising. Research at the student level has studied the experience of flow during music practice and has also studied the effect of pre-performance PP interventions on performance. For example, Fritz and Avsec (2007) reported that when undergraduate music students experienced flow during their music practice, this was linked to increases in positive emotions and life satisfaction. Broomhead et al. (2012) tested the effect of a PP teaching technique on the expressive performance of 155 junior high singers. The PP intervention involved a pre-performance routine of breathing and silent repetition of the words “bold”, “confident”, and “free”. Students in the PP group outperformed their junior high school singing peers who did not use the PP technique. More specifically, the PP group was rated by judges in a blind rating as having exhibited significantly superior expressive performance, dynamics, performance factors, and timing.

In addition to studying the effects of music teaching on student flow, research has also considered the effects of the music student–music teacher relationship on well-being. For example, Bakker (2012) extended the notion of flow in music students to also examine crossover experiences of flow between a student and teacher. In his study of 178 music teachers and 605 students from 16 different music schools, the results showed that flow between a student and teacher was contagious and induced in both parties a state of well-being.

Therefore, although the research on the applications of PP to music education^1^ is embryonic, the results obtained suggest that it is an area worthy of exploration. Furthermore, looking beyond the field of PP, the scholarship of music psychology has shown that studying and playing a musical instrument may impact motivational resources such as self-efficacy and intrinsic motivation (McPherson and Welsh 2012). Teaching which engenders positive self-efficacy and which progresses students from a state of extrinsic to intrinsic motivation, aids student musicians’ senses of autonomy, well-being, and competence (McPherson and Welch 2012). Conversely, teaching which inhibits the development of self-efficacy and undermines the development of intrinsic motivation, is related to music students becoming de-motivated and anxious (Craske and Craig 1984; Kivimäki 1995; Patston 2014). These findings suggest that a teaching model based on PP will be helpful for engaging studio music students.

## Studio Music Instruction in Schools

Instrumental music–education content, quality, and purpose vary greatly throughout the world. In some countries (e.g., Venezuela and Afghanistan) it is used as a model of social inclusion. In most first-world schools (e.g., Finland, Singapore, Australia, United Kingdom) it is a normal part of education (McPherson and Welch 2012). Ultimately, however, studio instrumental lessons, even if commenced in a classroom setting, become a discretionary activity. Students *choose* to study their instrument. Students are removed from timetabled classes, or learn before or after school in order to practise one-to-one in the studio with their teacher.^2^ Therefore engagement is critical. Teachers need students who want to come back week after week.

We suggest student engagement in studio music lessons can be boosted through a style of teaching which draws on their strengths and finds ways to facilitate deeper relationships with the students through an appreciative lens. We propose a framework which will complement existing pedagogic practice by improving students’ engagement and learning in the studio through providing them with a more positive mindset toward error correction and technical improvement.

## The PIMS Model

The PIMS model explicitly infuses key principles of PP into music studio teaching. It is important to understand that the model can be used iteratively and that teachers and students can go back and forth between the four steps. The model is not a formula to necessarily be followed step by step, each component can be used discretely as needed. The PIMS model can be integrated into specific methods-based programs, such as the Suzuki Method in instrumental playing or the Estill Method of vocal instruction, because it provides an underlying philosophical approach to pedagogy that may enhance student learning and engagement. This means that teachers can continue to use their existing pedagogic methods within a four-step philosophic process.

The PIMS model outlines four key processes that can facilitate a more positive instruction experience and foster greater learning:

- Start with positive priming exercises such as the “What Went Well?” (WWW) technique (Seligman et al. 2005), followed by students playing a piece they enjoy or believe they are good at (McPherson 2005).
- Connect students with their signature strengths (Brdar and Kashdan 2010) as deployed during their music practice.
- Use the positive pause by stopping when the student does something well (Kirschenbaum et al. 1982).
- Provide positive process praise by praising effort and technique, not just outcomes (Dweck 2007).

**Fig. 1:**
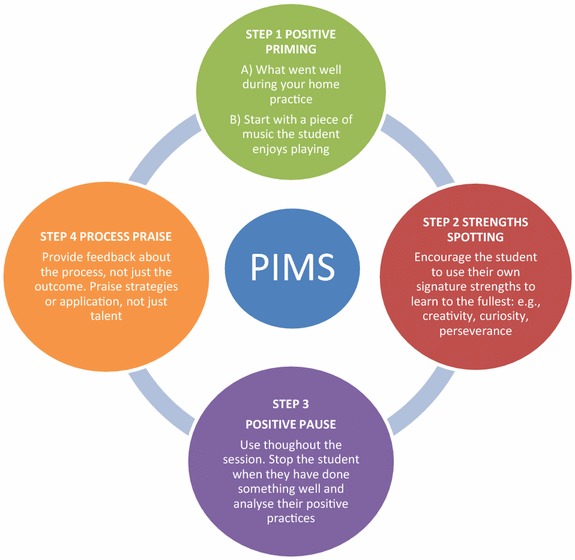
The positive instruction in music studios model

The intention of the model is to enable a more positive learning experience for music students. Each of the four steps in the model is outlined below (Fig. 1).

### Step 1: Positive Priming

Most conventional studio lessons follow a familiar path: a warm up followed by technical exercise, followed by fixing mistakes in repertoire, followed by playing a piece (Duke and Henninger 1998). By following a PP approach, we suggest that lessons can begin in a positive and affirming way. Positive priming sets up the mood and tone of the lesson which will follow. The WWW technique, which was first published by Seligman et al. (2005) and is now well-validated scientifically, asks people to focus on positive aspects of a situation. This technique has been significantly linked to well-being in adults (Seligman et al. 2005; Sin and Lyubomirsky 2009). Frederickson’s (2004) Broaden-and-Build theory has been used to explain why the WWW technique builds learning and well-being. The essence of the Broaden-and-Build theory is that positive emotions produce optimal function in individuals by sparking an urge to play and explore. The positive emotion generated means that people are more likely to build upon the task they are performing. The positive priming component of the PIMS should give students the spark to ignite and maintain a positive view of themselves and their music making.

There are many ways to begin a lesson positively. Under this model, each lesson begins with the question “What went well since our last lesson?” Students are encouraged to discuss anything from how they enjoyed playing a piece for their parents, to how they mastered a difficult phrase. This reinforces the positive nature of their music experiences beyond the studio. This question does not, indeed should not be specifically related to practice, but can relate to any aspect of music which the student has enjoyed or appreciated since their last lesson. An open-ended question may well elicit tastes, opinions, philosophies, or values the student holds in regard to music. Such knowledge can only assist teachers to understand their students and devise approaches and materials more effectively. From a student’s perspective the teacher is seen to have a broader interest in them as a person, not just a musician who practices. Confidence in their ability to express their opinions would be expected to raise self-esteem (Dweck 2007).

Following this brief discussion students are encouraged to begin their playing with a piece they enjoy. This may or may not be a piece chosen by the teacher. Many students play excerpts or pieces for enjoyment or fun at home, more than they practise what their teacher has directed (Hallam 2001). Often, they like to play one of the first pieces that they have mastered (McPherson 2005). Once again the student’s personal musical value system is validated. We believe that this process will engage the student’s mental, musical, and technical focus more rapidly than a demonstration of scales. Students who believe that they can contribute to the course and content of a lesson are more likely to be engaged and stimulated by the process (Blackwell et al. 2007). As the mind is engaged, and the music making has positive connotations from the very beginning of the lesson, the teacher can then focus on technical or repertoire aspects as they prefer, drawing on positive aspects which the student has demonstrated in the first piece they played. This feeling of experiencing and demonstrating mastery will aid the engagement process and sense of self-efficacy. It would also be expected that students in this state will be less likely to be anxious (Kirschenbaum et al. 1982; Kivimäki 1995).

### Step 2: Strengths Spotting

**Fig. 2:**
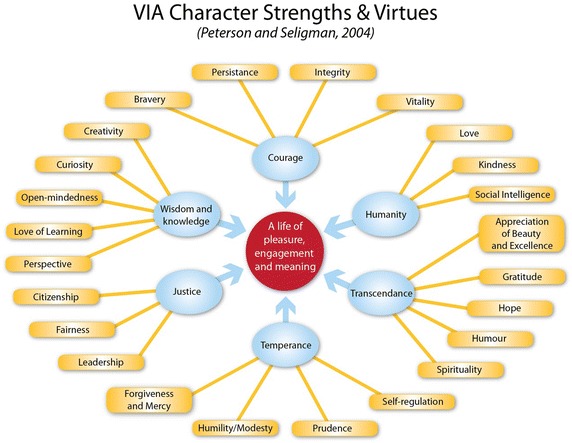
Character strengths

One of the prominent topics of study in PP is that of character strengths. In 2004 Professor Chris Peterson and Professor Martin Seligman developed the “Values in Action” (VIA) framework, which identified 24 character strengths. When developing the VIA framework, Peterson and Seligman’s research identified 24 character strengths that are universal to the human species. Each of the 24 strengths falls under one of six core virtues: wisdom, courage, humanity, justice, temperance, and transcendence, as shown in the diagram below (Fig. 2).

Character strengths are defined by Brdar and Kashdan (2010) as “pre-existing qualities that arise naturally, feel authentic, are intrinsically motivating to use and energizing.” (p. 151). Strengths of character are different to abilities and talents (such as intelligence, sporting prowess, music ability, or artistic ability). Not everyone may be high on talent, but everyone has character. It is important to stress that a character strength is different to a talent (Seligman 2002). A young musician may have a very good ear and the ability to mimic others (talent), but lack the character strengths such as curiosity, love of learning, and persistence to develop skills in sight reading. Research has shown that the character strengths model has been a successful learning tool in other academic disciplines such as Arts, Language (Seligman et al. 2005), English Literature (Barbieri et al. 2013), and Personal Development curriculum (Madden et al. 2010).

Studio music teachers can use the VIA character strengths framework to foster learning by being flexible in meeting their students’ needs. For example, the approach taken in a lesson toward a student who has a strength of prudence/caution will be different to someone whose signature strength is humour and playfulness. This is flexible, differentiated teaching and learning in action and more likely to enhance student engagement (Vaughn and Parsons 2013). Teachers who employ student-specific teaching strategies are more likely to maintain student interest and be more effective teachers (Darling-Hammond 2000; Duckworth et al. 2009). From a student’s perspective, their teacher will be demonstrating an active interest in the whole student, not just their musical and technical abilities.

### Step 3: Positive Pause (Teaching the Right, Not Just Correcting the Wrong)

Another aspect of conventional studio teaching is the “stop”. It is common for teachers to wait until they hear a mistake and then stop and correct (Duke and Simmons 2006). However, research by Kirschenbaum et al. (1982) found that pausing to reflect on positive technique in a sporting performance context significantly enhanced sporting performance and accounted for greater improvements in sporting performance than stopping to pause on incorrect technique and errors. The group of athletes who followed the traditional correctional technique and reflected on what went wrong in their sporting performance had significantly worse sporting outcomes than the group who engaged in the positive pause.

Under the PIMS model, music teachers are encouraged to ask the student to stop after they get something right, be it a rhythm, a pitch, a phrase, or a demonstration of technical facility. The students are told that lessons are about trying to find what they do well, so as to foster positive impact and learning from past success. This style of instruction not only builds student confidence, but also encourages students to explore solutions in independent practice from a strengths-based approach. It is a powerful tool which encourages an enjoyment of process, not a fixation on product. This step is not meant to replace that traditional technique of stopping students when they make a mistake, but, rather, we encourage studio instructors to consider the option of the positive pause as an effective learning strategy and for instructors to balance their use of the more corrective pause with the positive pause. We believe that this approach will encourage and develop an open mindset in students (Dweck 2007), with their analysis of positive aspects of study or performance informing their areas for improvement.

Given the high levels of performance anxiety and perfectionism in music education (Osborne and Kenny 2005; Patston 2010, 2014), a positive approach to problem solving will be helpful. Kirschenbaum et al. (1982) suggested that there are two mechanisms that may lead positive reflection to work: (1) attention and (2) reinforcement. In the music studio setting, attention may relate to a student reminding themselves of the correct fingering position before playing a phrase. Reinforcement may relate to repeating a phrase or passage which was executed correctly.

### Step 4: Process Praise

The concept of fixed- versus growth-mindset has recently received attention in the education literature (Coyle 2009; Dweck 2007; Heyman et al. 1992; Mueller and Dweck 1998). Students with a fixed mindset believe that they have a fixed level of intelligence or talent. The belief that musicians are born and not made has been a dominant view in music since the first attempts to predict music talent by Seashore in the 1920s (Lehmann and Gruber 2006). Conversely, those with a growth mindset believe that their talents can be developed, and that through application and accepting the challenges involved in developing new skills, students will be rewarded with improvement (Coyle 2009; Dweck 2007). Research shows that a growth mindset is more likely to be developed through the teacher’s use of process praise (Dweck 2007), which Dweck defines as praise for specific aspects put into the work, such as effort, resilience, and problem solving, rather than generic praise, such as “well-done” (Kamins and Dweck 1999).

The idea of a growth mindset has obvious applications in a music studio. Using the PIMS model, we encourage studio music teachers to specifically praise processes such as problem solving, technical development, determination, or concentration within a musical context. We predict that engagement followed by the motivation generated by a growth mindset will foster in students a love of music and a love of learning.

Students are faced with the challenges of developing their instrumental technique, learning the language of music, and then combining these to play and perform music. In music instruction, process praise would be given by the teacher for the way in which a student uses strategies, such as goal planning and execution, effort, application of specific technique or demonstration of engagement in the task. Such praise supports the notion of a flexible mindset and gives the message to the student that their music learning and performance can be improved. This is contrasted to person praise, or praising a student for a fixed quality such as intelligence or “natural talent”. In the music studio, a teacher may provide person praise and praise a student for their talent: “You are a naturally gifted musician”, or provide process praise such as: “Your tone in that phrase was even because you supported the breath throughout”. A number of studies (Aronson et al. 2002; Dweck 2007; Heyman et al. 1992; Mueller and Dweck 1998) have demonstrated that specific process praise results in higher levels of student motivation.

According to authors such as Kupers et al. (2014), and Madsen and Duke (1985), the most common phrase heard by teachers from their students after a performance or running through a piece of music is either negative or apologetic, or a combination of both. Students can easily become focused on errors and become harsh self-critics (Kupers et al. 2014; Patston 2014). Such mindsets can lead to the development of perfectionism or music performance anxiety (Patston 2014). By utilizing the PIMS model in lessons, teachers can then ask students to apply the model in their post-performance review. They can use positive priming before the performance and think about their character strengths. They can then reflect on the positive moments which occurred in the performance, before contextualising the performance with process praise in relation to their macro performance goals, rather than fixating on what are usually minor errors or lapses.

## Conclusion

The field of PP and its applications for education is growing. Researchers in the field of positive education have called for teachers to integrate the principles of PP into their teaching methods and approaches. We have answered this call with respect to studio music instruction and have put forward the PIMS model. The PIMS model offers an evidence-based four-step process based on the research evidence coming from positive psychology, as it applies to studio music education.

We suggest that PIMS has the potential to increase student engagement and learning in the music studio setting through beginning lessons in a positive way, affirming strengths, stopping to recognise achievement (in addition to correcting errors), and providing specific, process-related feedback.

## Footnotes

1 There has been considerable research conducted on the effect of listening to and playing music on psychological outcomes, but limited research on studio music teaching/music education.

2 Parents pay extra tuition for students to engage in studio music lessons.
